# Magnetically Tunable Vibration Transmissibility for Polyurethane Magnetic Elastomers

**DOI:** 10.3390/polym10010104

**Published:** 2018-01-22

**Authors:** Hiroyuki Endo, Shunsuke Kato, Mayuko Watanebe, Takehito Kikuchi, Mika Kawai, Tetsu Mitsumata

**Affiliations:** 1 Graduate School of Science and Technology, Niigata University, Niigata 950-2181, Japan; f16b003c@mail.cc.niigata-u.ac.jp (H.E.); f17b009g@mail.cc.niigata-u.ac.jp (S.K.); mikagoro@eng.niigata-u.ac.jp (M.K.); 2 ALCA, Japan Science and Technology Agency, Tokyo 102-0076, Japan; 3 Faculty of Engineering, Niigata University, Niigata 950-2181, Japan; t14s841j@mail.cc.niigata-u.ac.jp; 4 Faculty of Science and Technology, Oita University, Oita 870-1192, Japan; t-kikuchi@oita-u.ac.jp

**Keywords:** magnetic elastomer, magnetic gel, stimuli-responsive material, soft material, magnetorheological effect

## Abstract

The effect of a weak magnetic field on vibration transmissibility was investigated for magnetic elastomers with various volume fractions of magnetic particles. Polyurethane elastomers without magnetic particles exhibited a natural frequency at 53 Hz and were insensitive to a magnetic field of 60 mT. The natural frequency for magnetic elastomers with a volume fraction of 0.23 was 115 Hz at 0 mT, and increased to 134 Hz at 60 mT. The vibration transmissibility was independent of the magnetic field. A linear relation between the natural frequency and (*G*/*m*)^1/2^ was observed (*G*: storage modulus, *m*: mass), indicating that the observed vibration is basically described by a simple harmonic oscillation.

## 1. Introduction

Stimulus-responsive soft materials that are responsive to external stimuli such as temperature, pH, and electric fields have attracted considerable attention for use as soft actuators or sensors in next-generation devices. Magnetic elastomer is a stimulus-responsive soft material, and consists of polymeric matrices and magnetic particles. The viscoelastic property can be altered by the application of magnetic fields, and it has been widely studied experimentally and theoretically [1,2,3,4,5]. We have studied, thus far, the effect of magnetic fields on the viscoelastic property of magnetic elastomers, and have developed various types of magnetic elastomers [6,7,8,9].

Viscoelastic materials such as gels, rubbers, or elastomers can be applied to vibration absorbers. Vibration absorbers in commercial use are able to attenuate the vibration only around their natural frequencies. However, machines with motors generate vibrations at various frequencies, depending on the rotation rates. Therefore, a large number of absorbers is needed to erase vibrations with various frequencies. It is generally known in vibration engineering that a vibrating object demonstrates a natural frequency on the frequency spectrum of vibration transmissibility, and the natural frequency’s relationship to the elastic modulus of the vibration object can be expressed by means of the following relation [10],

(1) $$\frac{\Delta f}{f_{0}}\propto \sqrt{1+\frac{\Delta G}{G_{0}}}-1$$

here, Δ*G*’/*G*’_0_ is the fractional change in elastic modulus and Δ*f*/*f*_0_ is the corresponding fractional change in natural frequency. This means that magnetic elastomers are useful for application in active dampers, because the natural frequency can be continuously changed by magnetic field strength. Komatsuzaki et al. have fabricated a dynamic vibration absorber using magnetorheological elastomers that demonstrates the ability of broadband vibration control [11,12]. They reported that the natural frequency shifted from 60 to 250 Hz by applying an electric current of 3.5 A [11]. Nguyen et al. developed a vibration isolator with a frequency shift of ~8 Hz by applying a magnetic field of 218 mT [13]. Fu et al. have also developed a semi-active/fully-active hybrid isolator using magnetorheological elastomer and piezoelectric material. They found that the natural frequency can be shifted by approximately 20 Hz by applying an exciting current of 1 A to the electromagnet [14].

In this study, we report the effect of a magnetic field on the vibration absorbing properties for magnetic elastomers with various volume fractions of magnetic particle.

## 2. Materials and Methods

### 2.1. Synthesis of Magnetic Elastomers

Polyurethane elastomers and magnetic elastomers were synthesized by a prepolymer method. Polypropylene glycols (*M*_w_ = 2000, 3000), prepolymer cross-linked by tolyrene diisocyanate (Wako Pure Chemical Industries. Ltd., Osaka, Japan), a plasticizer (dioctyl phthalate, DOP, Wako Pure Chemical Industries. Ltd., Osaka, Japan), and carbonyl iron (CI, CS grade, BASF SE., Ludwigshafen am Rhein, Germany) particles were mixed using a mechanical mixer for several minutes. The median diameter of CI particles was 7.0 ± 0.2 μm, as determined by a particle size analyzer (SALD-2200, Shimadzu Co. Ltd., Kyoto, Japan). The saturation magnetization for CI particles was evaluated to be 245 emu/g by SQUID magnetometer (MPMS, Quantum Design Inc., San Diego, CA, USA). The mixed liquid was poured in a silicon mold and cured in an oven for 20 min at 100 °C. The weight concentration of DOP was defined by the ratio of DOP to the matrix without magnetic particles and it was fixed at 50 wt %; DOP/(DOP + matrix). The weight fraction of magnetic particles (CI-CS) was varied up to 70 wt %; CI/(CI + matrix), which corresponds to a volume fraction of 0.23.

### 2.2. Vibration Experiments

**Figure 1:**
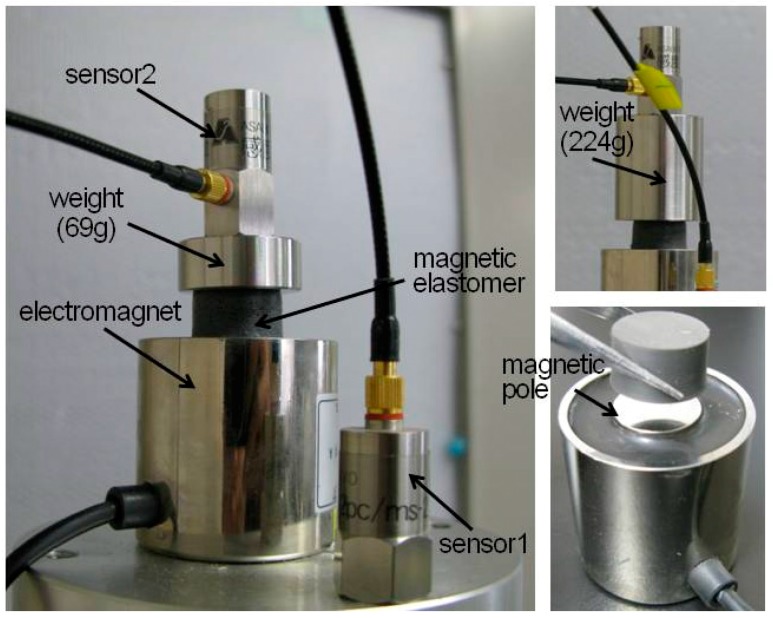
Photographs of the experimental set-up for the vibration measurements for polyurethane and magnetic elastomers under magnetic fields.

Figure 1 shows a photograph of the experimental set-up for the vibration measurements of polyurethane elastomers and magnetic elastomers. Vibration experiments were carried out using a vibration exciter (SW-2015, Asahi factory Co. Ltd., Hino, Japan) at room temperature. The acceleration was measured by a piezo electric acceleration sensor (P51C, San-ei Instruments Inc., Toshima, Japan) with varying the frequency from 5 to 300 Hz at room temperature. We measured the frequency spectra of transmissibility for three different samples obtained from different batches, and the averaged value and standard error are shown. The sample was a cylinder with a diameter of 20 mm and a length of 10 mm. The transmissibility τ was calculated from the following equation [15,16],

(2) $$\tau =\frac{\alpha_{2}}{\alpha_{1}}=\sqrt{\frac{1+{\left(\tan\delta \right)}^{2}}{{\left(1-{\left(\frac{f}{f_{0}}\right)}^{2}\right)}^{2}+{\left(\tan\delta \right)}^{2}}}$$

here, α_1_ and α_2_ are the acceleration measured by sensor 1 (vibrating stage of exciter) and sensor 2 (after attenuation by magnetic elastomer), respectively. *f* and *f*_0_ are the frequency and natural frequency, respectively. tanδ is the loss factor of elastomers. A cylindrical electromagnet (FSGP-40, Fujita Co. Ltd., Kuwana, Japan) with a weight of 300 g was used for generating magnetic fields. The electric potential and electric current were 22.3 V and 0.23 A, respectively, when a magnetic field of 60 mT was applied. The magnetic field strength was measured on the top and the center of the electromagnet by a Hall sensor (TM-601, Kanetec Co. Ltd., Ueda, Japan). An enlarged photograph of the electromagnet is also shown in Figure 1. There is no yoke on the electromagnet for a closed circuit of magnetic flux; therefore, the magnetic field strength is extremely weak. If an electromagnet generating uniform magnetic fields were used, the magnetic effect on the vibration property would be enhanced. Two kinds of weight with masses of 69 and 224 g were used to clear the effect of mass on the natural frequency or transmittance. The strain was calculated to be 1.4 × 10^−2^–5.1 × 10^−2^ for a loading weight of 69 g and 4.7 × 10^−2^–1.7 × 10^−1^ for a loading weight of 224 g using the values of storage modulus (=3*G*’). The strain dependence for magnetic elastomers was similar to that previously reported [9]. Most of magnetic elastomers studied here exhibited the nonlinear viscoelasticity; however, only samples without magnetic particles and φ= 0.03 demonstrated the linear viscoelasticity at whole strains.

### 2.3. Dynamic Viscoelastic Measurements

To clear the viscoelastic properties of polyurethane and magnetic elastomers, the dynamic viscoelastic measurements were carried out using a rheometer (MCR301, Anton Paar Pty. Ltd., Graz, Austria) at 20 °C. The strain was constant at 10^−4^, and the frequency was constant at 1 Hz. Although the frequency is different from that in the vibration experiment, the viscoelastic data are listed in Table 1, as a reference. An electric current with 0.33 A was used for the rheological measurement which corresponds to a magnetic field of 60 mT. The sample was a disk 20 mm in diameter and 1.5 mm thick.

## 3. Results and Discussion

**Figure 2:**
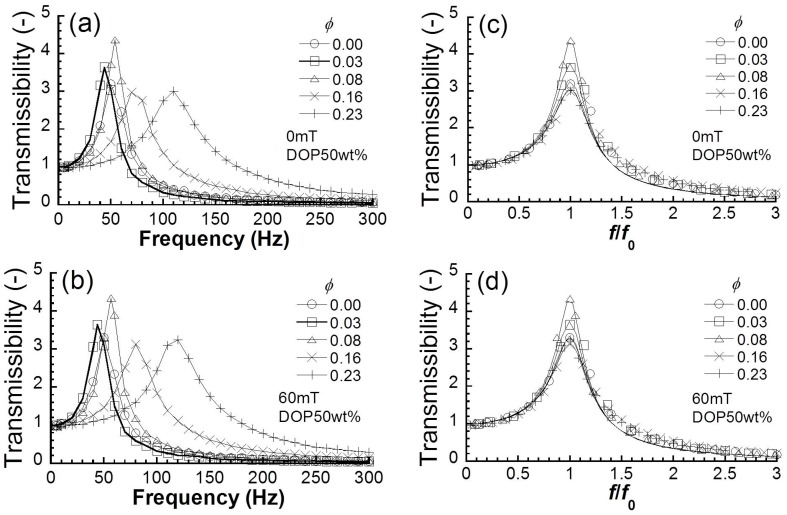
Frequency spectra of transmissibility for magnetic elastomers with various volume fractions of magnetic particles at (**a**) 0 mT and (**b**) 60 mT. Normalized frequency spectra for magnetic elastomers at (**c**) 0 mT and (**d**) 60 mT (loading weight 69 g).

Figure 2a exhibits the frequency spectra of transmissibility at 0 mT for magnetic elastomers with various volume fractions of magnetic particles. The natural frequency for magnetic elastomers without magnetic particles was seen at approximately 53 Hz. The frequency spectra shifted to higher frequencies with an increasing volume fraction of magnetic particles. Figure 2b shows the frequency spectra of transmissibility at 60 mT for magnetic elastomers with various volume fractions of magnetic particles. The frequency spectra for magnetic elastomers at 60 mT were similar to those at 0 mT. Figure 2c,d demonstrates the frequency spectra of transmissibility normalized by the natural frequency at 0 and 60 mT, respectively. The peaks of transmissibility for magnetic elastomers with high volume fractions of magnetic particles were slightly wider than those for polyurethane elastomers independently of the magnetic field. This means that the loss factor of magnetic elastomers is higher than that of polyurethane elastomers. The loss factor of magnetic elastomers determined by the fitting of Equation (2) is also listed in Table 1. It was also seen that the loss factor at 0 mT increased with the volume fraction of magnetic particles, and was almost insensitive to the magnetic field.

**Figure 3:**
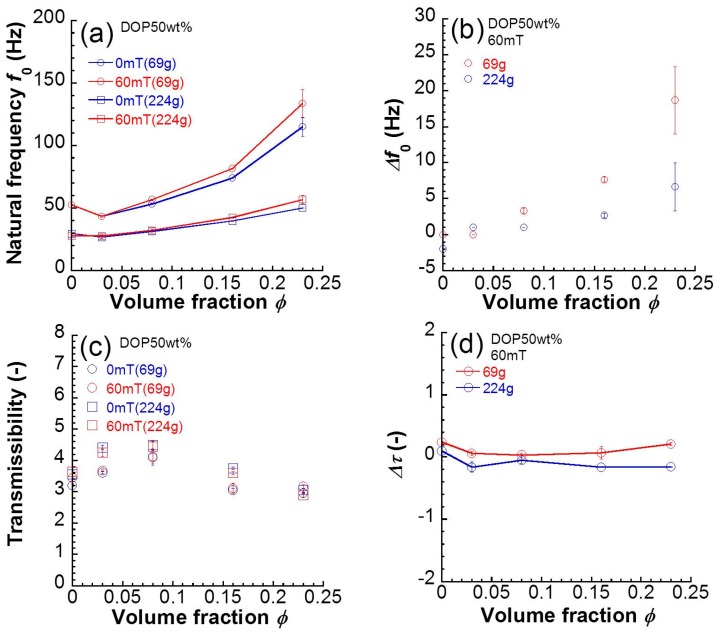
Relationship between (**a**) natural frequency or (**c**) transmissibility at 0 and 60 mT and volume fraction of magnetic particles. Change in (**b**) natural frequency or (**d**) transmissibility and volume fraction of magnetic particles.

Figure 3a shows the relationship between the natural frequency and the volume fraction of magnetic particles for magnetic elastomers in the absence and presence of a magnetic field of 60 mT. The natural frequency for polyurethane elastomers without magnetic particles was 53 Hz. Both at 0 and 60 mT, the natural frequency for magnetic elastomers increased with the volume fraction of magnetic particles. It was clear that the natural frequencies at 60 mT for magnetic elastomers with high volume fractions of magnetic particles were higher than those at 0 mT. Figure 3b exhibits the relationship between the change in natural frequency and the volume fraction of magnetic particles. The change in natural frequency significantly increased with the volume fraction of magnetic particles. At φ = 0.23, the change in natural frequency reached 23 Hz. Figure 3c depicts the relationship between transmissibility and the volume fraction of magnetic particles for magnetic elastomers. The transmissibility for polyurethane elastomers without magnetic particles was 3.2. Both at 0 and 60 mT, the transmissibility for magnetic elastomers was independent of the volume fraction of magnetic particles. There was no clear difference in the transmissibility for magnetic elastomers at 0 and 60 mT. Figure 3d demonstrates the relationship between the change in transmissibility and the volume fraction of magnetic particles. The change in transmissibility did not alter by varying the volume fraction of magnetic particles. No magnetic field effect was observed in the transmissibility for magnetic elastomers. The data for natural frequency and transmissibility when a loading weight of 224 g was applied are also shown in Figure 3. The mechanism of the frequency shift by weak magnetic fields is not yet clear; however, it could be that large strains induced by the heavy weight reduce the frequency shift.

**Figure 4:**
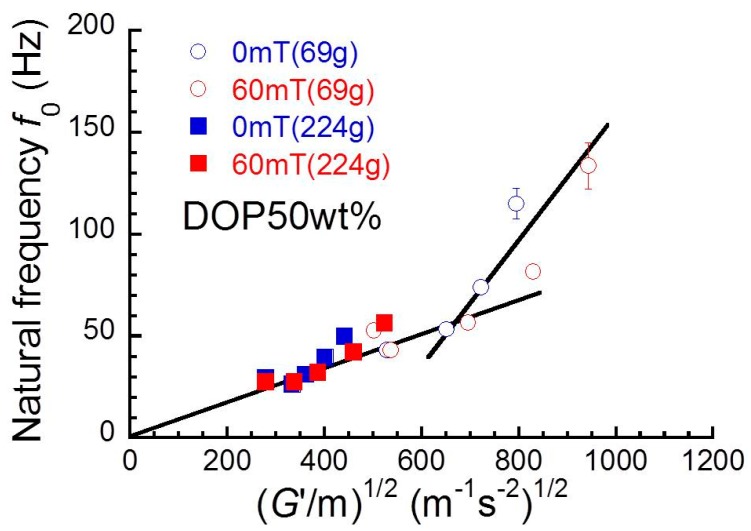
Relationship between natural frequency and (*G*’/*m*)^1/2^ in Equation (3) for all magnetic elastomers under two different loads.

Figure 4 shows the relationship between natural frequency and (*G*’/*m*)^1/2^ for magnetic elastomers with various volume fractions of magnetic particles. If the vibration observed here is a simple harmonic motion, the natural frequency relates to the elastic modulus *G*’ and mass *m* of the vibration object as the following relation [15,16],

(3) $$f_{0}\propto \sqrt{\frac{G^{\prime }}{m}}.$$

As seen in the plot, the natural frequency for most samples measured at 224 g was well explained by Equation (3); however, the natural frequency measured at 69 g deviated from the line at around ~650 N^1/2^·m^−1^·kg^−1/2^. This means that magnetic elastomers show the linear viscoelastic response at high load. In general, the nonlinear viscoelasticity is significant at high strains (i.e., at high load). This contradiction is not yet clear; however, the natural frequency might be strongly influenced by structures such as particle network at 0 mT or chain structure at 60 mT. This is because that their structures are collapsed under high strains. To fully elucidate the relation between natural frequency and nonlinear viscoelasticity, viscoelastic measurements at high frequency at around 100 Hz are necessary, and this will be reported in a subsequent paper.

**Figure 5:**
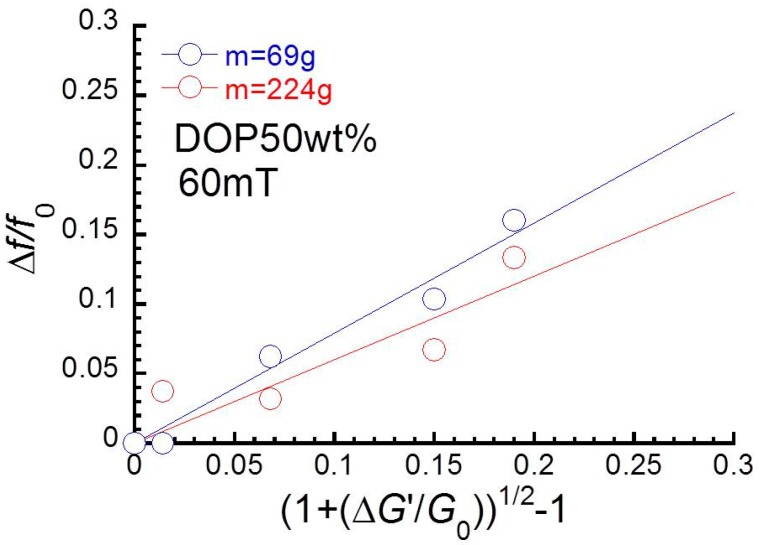
Relationship between the change in natural frequency and the value calculated from Equation (1) for all magnetic elastomers under two different loads.

Figure 5 depicts the relationship between the change in natural frequency and the value calculated from Equation (1) at two loading weights. It was clear that the change in natural frequency was in proportional to a function of the change in storage modulus, i.e., $\sqrt{1+\Delta G^{\prime }/G_{0}}-1$. It was also found that the slope with a weight of 224 g was lower than that at 69 g.

## 4. Conclusions

We investigated the effect of magnetic field on the vibration absorbing properties of magnetic elastomers. A natural frequency was observed on the frequency spectrum at frequencies below 150 Hz. A magnetic effect was observed for magnetic elastomers with a volume fraction of 0.23 whereby the natural frequency shifted to higher frequency by 23 Hz with the application of a magnetic field of 60 mT. This means that the vibration can be controlled by such small magnetic fields. A plot of natural frequency and (*G*’/*m*)^1/2^ revealed that the observed vibration can be basically explained by a simple harmonic motion. However, the material deviates from this simple harmonic model at low loads, warranting further study. The total weight of the device containing the electromagnet is only 320 g. We firmly believe that magnetic elastomers are useful for active dampers with compact size.

## Figures and Tables

**Table 1:** Loss factor tanδ determined from transmissibility spectra (loading weight 69 g), storage modulus *G’,* loss modulus *G’’*, and loss factor tanδ determined from rheological measurement for polyurethane and magnetic elastomers.

φ ^1^	Fitting		Rheometer					
	tanδ ^2^		G’ ^3^ (kPa)		G” ^4^ (kPa)		tanδ ^5^	
	0 mT	60 mT	0 mT	60 mT	0 mT	60 mT	0 mT	60 mT
0.00	0.32	0.29	17	17	2.3	2.3	0.13	0.13
0.03	0.28	0.28	20	20	1.9	1.9	0.10	0.10
0.08	0.26	0.26	29	33	2.8	3.1	0.09	0.09
0.16	0.33	0.32	36	48	3.5	4.3	0.10	0.09
0.23	0.36	0.33	44	61	4.0	5.9	0.09	0.09

^1^ Volume fraction of magnetic particles; ^2^ Loss factor; ^3^ Storage modulus at 1 Hz; ^4^ Loss modulus at 1 Hz; ^5^ Loss factor at 1 Hz.
